# Inflammatory Biomarkers and Post-Intensive Care Syndrome: A Prospective Cohort Study

**DOI:** 10.3390/diagnostics16152361

**Published:** 2026-07-27

**Authors:** Mateusz Szczupak, Jacek Kobak, Jolanta Wierzchowska, Jakub Wiśniewski, Marek Konop, Sabina Krupa-Nurcek

**Affiliations:** 1 Department of Anaesthesiology and Intensive Therapy of the Nicolaus Copernicus Hospital in Gdańsk, Nowe Ogrody 1-6 Street, 80-803 Gdańsk, Poland; szczupak.mateusz@icloud.com (M.S.); jwierzchwoska@wss.gda.pl (J.W.); 2 Department of Otolaryngology, Faculty of Medicine, Medical University of Gdańsk, Sklodowskiewj-Curie 3a Street, 80-210 Gdańsk, Poland; 3 Department of Otolaryngology, University Clinical Center, Mariana Smoluchowskiego 17 Street, 80-214 Gdańsk, Poland; 4 Department of Neurosurgery, Copernicus Hospital in Gdańsk, Nowe Ogrody 1-6 Street, 80-803 Gdańsk, Poland; 5 Laboratory of the Center for Preclinical Research, Department of Experimental Physiology and Pathophysiology, Medical University of Warsaw, 02-106 Warsaw, Poland; 6 Department of Surgery, Faculty of Medicine, Collegium Medicum, University of Rzeszów, Waclawa Kopisto 2A Street, 35-959 Rzeszów, Poland

**Keywords:** post-intensive care syndrome, ICU, C-reactive protein, procalcitonin, interleukin-6, inflammation, biomarkers

## Abstract

**Background and Objective:** Post-Intensive Care Syndrome is a multidimensional sequela of critical illness that includes physical, cognitive, and psychological impairments after intensive care unit discharge. Systemic inflammation has been proposed as one potential mechanism contributing to selected PICS domains, but the direction, timing, and clinical relevance of this relationship remain uncertain. This study assessed whether serial concentrations of C-reactive protein, procalcitonin, and interleukin-6 were associated with PICSQ severity in ICU survivors. **Materials and Methods:** This prospective single-center cohort study included 267 adult ICU patients in the primary complete case analysis. CRP, PCT, and IL-6 were measured at predefined time points during hospitalization. PICS severity was assessed using the Post-Intensive Care Syndrome Questionnaire at ICU discharge and at 2 and 3 months after discharge. The primary endpoint was total PICSQ severity at 3 months. Correlation analyses, threshold-based group comparisons, and logistic and multivariable linear regression models adjusted for age, sex, reason for ICU admission, and length of hospitalization were performed. Secondary and domain-specific analyses were considered exploratory. **Results:** Correlation analyses did not show a consistent association between inflammatory biomarkers and total PICSQ severity at ICU discharge, 2 months, or 3 months. Weak inverse associations were observed between selected CRP, PCT, and IL-6 measurements and cognitive domain scores, indicating lower cognitive impairment scores among patients with higher biomarker values in some analyses. These findings were not consistent across time points and should be interpreted cautiously. In unadjusted threshold-based analyses at 3 months, selected associations were observed for CRP after one week, PCT on day 4, and IL-6 on days 2 and 4, mainly in the psychological domain. In fully adjusted linear regression models, none of the selected biomarker thresholds remained statistically significant at *p* < 0.05. In adjusted linear regression, PCT ≥ 2 ng/mL on day 4 showed a borderline association with the total PICSQ score at 3 months, with β = 0.44, 95% CI −0.07 to 0.94, *p* = 0.089, N = 267. IL-6 > 10 pg/mL on day 2 showed a borderline association with psychological domain severity at 3 months, with β = 0.46, 95% CI from −0.005 to 0.92, *p* = 0.052. In adjusted logistic regression, IL-6 > 10 pg/mL on day 2 was associated with higher odds of psychological domain score ≥ 4 at 3 months, with adjusted OR 3.61, 95% CI 1.26 to 10.37, *p* = 0.017, N = 266. No consistent association was observed for the physical domain. **Conclusions:** In this exploratory cohort, routinely available inflammatory biomarkers were not consistently associated with global PICSQ severity across all assessment time points. Selected time-dependent and domain-specific associations, particularly involving PCT and IL 6 at 3 months, may indicate a possible link between inflammatory activation and later psychological PICS burden. These findings do not establish causality or predictive performance and require external validation before CRP, PCT, or IL 6 can be used for PICS risk stratification.

## 1. Introduction

Post-intensive care syndrome (PICS) is an increasingly recognized, multifaceted consequence of critical illness and intensive care unit (ICU) hospitalization. It encompasses new or worsening cognitive impairment, physical limitations, and mental health problems that emerge after ICU discharge and persist for months and sometimes years [1,2,3,4]. As mortality rates among ICU patients decline, the number of survivors who require further evaluation and care due to the long-term consequences of intensive care is increasing [1,2]. Epidemiological data indicate that cognitive impairment after ICU stay occurs with variable frequency, particularly in patients with a history of sepsis, acute respiratory distress syndrome (ARDS), or prolonged mechanical ventilation [3,4,5,6]. Psychological symptoms—such as depression, anxiety, or post-traumatic stress disorder are also frequent after critical illness and may coexist with cognitive or physical impairment [2,4,7]. Physical limitations, muscle weakness, and loss of functional independence are other major components of PICS and contribute to reduced quality of life, rehospitalization, and social and economic burden [7,8,9,10]. These consequences are associated with a significant decline in quality of life, increased hospital readmissions, and significant social and economic costs [1,2,8]. The pathogenesis of PICS is multifactorial. Age, comorbidities, clinical severity, sepsis, multi-organ failure, hypoxia, prolonged immobilization, sedation, and delirium in the intensive care unit all play significant roles [2,9,10,11]. However, increasing attention is being paid to the systemic inflammatory response as a potential common mechanism underlying long-term neurological, psychological, and functional sequelae.

Persistent inflammatory activation may be associated with neuroinflammation, endothelial injury, microcirculatory dysfunction, mitochondrial impairment, muscle catabolism, and delayed tissue recovery [12,13,14,15]. These mechanisms are biologically plausible but were not directly measured in the present cohort; therefore, they should be interpreted as explanatory hypotheses rather than mechanisms demonstrated by this study. Prior ICU survivor studies have linked inflammatory biomarkers, including IL-6 and IL-10, with neuropsychiatric outcomes and cognitive dysfunction [12,13,14]. In addition, prospective data in ICU survivors suggest that IL-6 may be independently associated with depression, supporting a rationale for examining the psychological domain rather than focusing only on cognition [12].

C-reactive protein (CRP), procalcitonin (PCT), and interleukin-6 are among the most frequently measured biomarkers of the inflammatory response in critically ill patients. These markers are widely available, relatively inexpensive, and routinely utilized in daily clinical practice. Despite the biological plausibility of their potential association with the development of PICS, data on their prognostic value for long-term sequelae following intensive care remain limited and inconclusive [14,15,16,17].

Given the growing number of patients surviving ICU treatment, identifying simple, accessible biomarkers for PICS risk could be of significant clinical importance, enabling earlier implementation of monitoring, rehabilitation, and follow-up care after discharge.

## 2. Aim of Study

The aim of this study was to assess the association between selected inflammatory parameters, specifically CRP, PCT, and IL-6 concentrations, and the severity of Post-Intensive Care Syndrome in patients hospitalized in an intensive care unit.

A secondary objective was to evaluate whether changes in inflammatory markers during hospitalization were associated with subsequent severity in the physical, cognitive, and psychological PICSQ domains at ICU discharge and at 2 and 3 months after ICU discharge.

Because previous evidence is limited and biologically heterogeneous, the main hypothesis was formulated as an association hypothesis rather than as a strictly directional predictive hypothesis. Higher inflammatory marker concentrations were expected to be associated with greater PICSQ severity in selected domains, but inverse or domain-specific associations were considered possible exploratory findings requiring cautious interpretation.

The study did not aim to estimate the incidence or occurrence of PICS as a binary outcome. All analyses should therefore be interpreted as analyses of PICS severity rather than PICS incidence.

## 3. Materials and Methods

### 3.1. Study Design

A prospective, observational, single-center cohort study was conducted among adult patients hospitalized in a multidisciplinary Intensive Care Unit. The aim of the study was to assess the relationship between selected inflammatory markers measured during ICU stay and the subsequent severity of Post-Intensive Care Syndrome following completion of intensive care treatment. Recruitment was conducted continuously among patients who met the eligibility criteria.

### 3.2. Study Population

During the study period, 490 adult ICU patients were screened for eligibility. Patients were eligible for inclusion if they were aged 18 years or older, required intensive care admission, and were eligible for clinical and questionnaire-based assessment during the observation period. Patients were excluded in the presence of severe primary central nervous system injury precluding assessment, a pre-hospitalization diagnosis of severe neuropsychiatric disorder significantly affecting cognitive or mental assessment, active alcohol or psychoactive substance dependence, refusal to participate, early death before assessment, or loss to follow-up or unavailability at the 3-month assessment.

**Figure 1:**
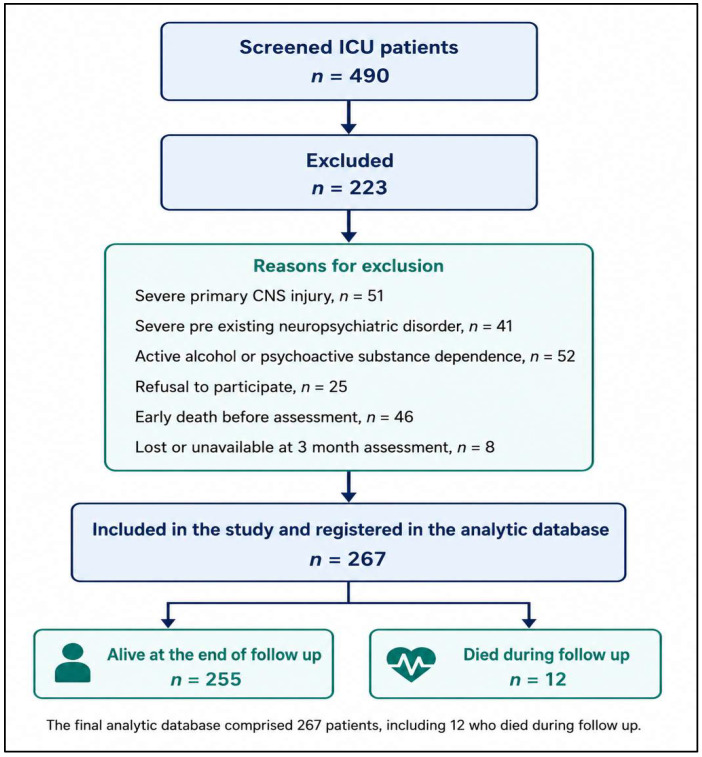
Participant flow diagram.

After application of the eligibility criteria, 267 patients were included in the study and registered in the analytic database. Among these patients, 255 were alive at the end of follow-up, and 12 died during follow-up. These deaths were recorded within the final analytic database and were not subtracted from the total cohort size. Therefore, the final analytic database comprised 267 patients, including 12 patients who died during follow-up. Participant flow is shown in Figure 1.

For secondary analyses, the effective sample size varied depending on the availability of biomarker measurements and PICSQ domain assessments at each time point. Therefore, the number of observations included in each analysis and the subgroup denominators are reported in the corresponding main or Supplementary Table. Patients with missing biomarker data or missing questionnaire assessments for a given endpoint were excluded from the corresponding complete case analysis. Missing data were not imputed. Complete case analyses were performed separately for each biomarker, endpoint, and assessment time point.

**Inclusion Criteria:**

- Age ≥ 18 years;
- ICU admission requiring intensive care;
- Ability to obtain informed consent from the patient or a legal representative;
- Ability to conduct clinical and questionnaire-based assessments during the observation period.

**Exclusion Criteria:**

- Age < 18 years;
- Severe primary central nervous system injury precluding assessment;
- Pre-hospitalization diagnosis of severe neuropsychiatric disorders significantly affecting cognitive or mental assessment;
- Active alcohol or psychoactive substance dependence;
- Refusal to participate in the study;
- Death occurred early in the hospitalization period, precluding further observation.

Loss to follow-up was minimized by scheduling follow-up assessments at discharge and by allowing standardized telephone assessment when an in-person visit was not feasible. Missing data were not imputed. Complete case analyses were performed separately for each biomarker, endpoint, and assessment time point.

Participant flow is shown in Figure 1.

### 3.3. Collection of Clinical Data

Clinical and laboratory data were collected prospectively during the patients’ hospitalization in the Intensive Care Unit (ICU). The parameters assessed included demographic data, the reason for ICU admission, length of hospitalization, and the results of serial measurements of inflammatory markers.

### 3.4. Laboratory Measurements

Selected biochemical markers of inflammation—routinely measured in patients hospitalized in the Intensive Care Unit—were subjected to analysis; these included C-reactive protein, procalcitonin, and interleukin-6. Laboratory results were retrieved from the patients’ electronic medical records, and, for analysis, measurements obtained at consecutive time points during hospitalization—in accordance with the adopted study schedule—were included.

CRP concentrations were determined using an immunoturbidimetric method; PCT concentrations were determined using an electrochemiluminescence method; and IL-6 concentrations were determined using an enzyme-linked immunosorbent assay (ELISA).

The hospital laboratory reference ranges were adopted as reference values. For CRP, concentrations of 5 mg/L or lower were considered within the reference range, whereas values above 5 mg/L were classified as elevated. For PCT, concentrations of 0.5 ng/mL or lower were considered within the reference range; values between 0.5 ng/mL and 2 ng/mL were treated as an intermediate category; and values of 2 ng/mL or higher were treated as marked PCT elevation. For IL-6, values of 7 pg/mL or lower were considered within the laboratory reference range, whereas values above 10 pg/mL were used in exploratory threshold-based analyses. Biomarkers were also analyzed as continuous variables.

PCT thresholds of ≥2 ng/mL and IL6 > 10 pg/mL were used as analytical thresholds and were not intended to diagnose bacterial infections or systemic inflammatory disease. These thresholds were predetermined before final analysis.

### 3.5. Assessment of Post-Intensive Care Syndrome

Post-Intensive Care Syndrome was assessed using the Post-Intensive Care Syndrome Questionnaire, a self-report instrument developed to evaluate post-intensive care morbidity in ICU survivors. The questionnaire assesses three core domains of PICS: cognitive impairment, physical impairment, and psychological impairment. The version used in the present study consisted of 18 items, with 6 items assigned to each domain. Items 1 to 6 assessed cognitive impairment, items 7 to 12 assessed physical impairment, and items 13 to 18 assessed psychological impairment.

Each item was rated on a four-point Likert-type scale from 0 to 3, where 0 indicated absence of the symptom, and 3 indicated the highest frequency or severity of the symptom. The possible score range was therefore 0 to 18 points for each domain and 0 to 54 points for the total PICSQ score. Higher domain and total scores indicated greater severity of post-intensive care impairment.

PICSQ assessments were performed at ICU discharge, 2 months after ICU discharge, and 3 months after ICU discharge. The total PICSQ score and the cognitive, physical, and psychological domain scores were analyzed as continuous severity outcomes. The primary endpoint was the association between inflammatory biomarker concentrations during ICU hospitalization and total PICSQ severity at 3 months after ICU discharge. Domain-specific analyses at ICU discharge, 2 months, and 3 months were considered secondary and exploratory.

For exploratory logistic regression analyses, psychological domain severity at 3 months was additionally dichotomized using a psychological domain score of ≥4 as the threshold for higher psychological symptom burden. This threshold was used as an analytical severity threshold and should not be interpreted as a validated diagnostic cutoff for PICS.

### 3.6. Endpoints

The primary endpoint involved the association between inflammatory marker concentrations during hospitalization and the total severity of PICSQ 3 months after ICU discharge.

Secondary endpoints addressed:

- the association of biomarkers with PICSQ domain scores;
- the relationship between the persistence of elevated markers and PICS severity;
- changes in PICS severity between consecutive follow-up time points.

### 3.7. Statistical Analysis

Statistical analysis was performed using Statistica 13.3 PL software. Quantitative variables were summarized as mean and standard deviation, median, range and quartiles. Qualitative variables were summarized as counts and percentages. Variable distributions were assessed using histograms, the Shapiro–Wilk test, skewness, and kurtosis.

Analyses were conducted using a complete case approach for each endpoint and biomarker time point. The number of observations included in each analysis was treated as the effective sample size and is reported in the corresponding main or Supplementary Table, including subgroup denominators for threshold-based comparisons.

The primary analysis was an adjusted linear regression model assessing the association between selected biomarker exposures during ICU hospitalization and total PICSQ score at 3 months. Analyses of individual PICSQ domains, earlier follow-up time points, repeated biomarker time points, threshold-based group comparisons, and logistic regression models for psychological domain score ≥ 4 at 3 months were considered exploratory.

Pearson correlation was used when variables met the assumptions of approximate normality. Spearman rank correlation was used for non-normally distributed or ordinal variables. Between group comparisons were performed using Student t test or analysis of variance when distributional assumptions were met, and the Mann–Whitney U test or Kruskal–Wallis test when distributions were skewed or subgroup sizes were small. For two group comparisons, mean differences with 95% confidence intervals and Hedges g effect sizes were reported where appropriate.

Linear regression models used continuous PICSQ total or domain scores as dependent variables. Logistic regression models used psychological domain score ≥ 4 at 3 months as the dependent variable. Adjusted models included age, sex, reason for ICU admission, and length of hospitalization. These variables were selected a priori as basic clinical covariates. Because the length of hospitalization may also lie along the causal pathway between inflammatory activation and subsequent PICS severity, models that include this variable should be interpreted as conservatively adjusted estimates.

The study used an available prospective cohort, and no a priori sample size calculation was performed. With 267 complete observations, the cohort had approximately 80% power to detect a two-sided correlation of about r = 0.17 at alpha = 0.05 for a single primary association. This calculation does not account for the substantially larger number of secondary and exploratory comparisons.

No formal correction for multiple comparisons was applied. Therefore, secondary and exploratory *p* values should be interpreted descriptively, and isolated statistically significant findings, particularly those close to 0.05, should not be overinterpreted. Correlation coefficients below 0.20 in absolute value were interpreted as weak and not considered clinically meaningful unless consistent across time points or models.

### 3.8. Ethical Considerations

The study was conducted in accordance with the principles of the Declaration of Helsinki. Approval was obtained from the relevant Bioethics Committee (KB 6A/24), and all study participants—or their legal representatives—provided informed consent to participate in the study.

## 4. Results

### 4.1. Demographic Analysis of the Study Cohort

The final analysis included 267 patients hospitalized in the Intensive Care Unit. The mean age of the study participants was 52.6 ± 13.9 years, and men constituted 58.8% of the population (n = 157). The largest age group consisted of patients aged 61–70 years (33.7%).

The most common reasons for admission to the ICU were circulatory failure (34.8%), respiratory failure, acute renal failure, and gastrointestinal bleeding. The mean duration of hospitalization was 19.9 ± 9.3 days.

Hospital survival was recorded in 255 patients (95.5%), while 12 patients died during treatment.

A detailed characterization of the study population is presented in Table 1.

### 4.2. Analysis of the Characteristics of PICS Development in the ICU Patient Group

For the group of patients hospitalized in the Intensive Care Unit, PICSQ total and domain scores were summarized descriptively and used as dependent variables in subsequent analyses. Distributional indices were reviewed before selecting statistical methods. Most skewness and kurtosis values were within the predefined range of −1 to 1; however, selected endpoints showed deviations from this range, including total PICSQ at 3 months and the psychological domain score at 3 months. Therefore, distributional assumptions were interpreted with caution, and non-parametric or regression-based approaches were used when appropriate. Descriptive statistics are presented in Table 2.

### 4.3. Analysis of the Correlation Between Selected Inflammatory Parameters and Post-Intensive Care Syndrome

To assess the association between serial inflammatory biomarker levels and PICSQ symptom severity, correlation analysis was performed between CRP, PCT, and IL-6 levels measured at fixed time points during hospitalization and PICSQ total and domain scores assessed at ICU discharge, 2, and 3 months after discharge. Because the analysis included multiple biomarkers, repeated time points, and multiple PICSQ scores, the results were interpreted descriptively and exploratorily. Detailed data are presented in Table 3.

Correlation analyses did not show a consistent association between inflammatory biomarkers and total PICSQ severity across ICU discharge, 2-month, and 3-month assessments. Several statistically significant correlations were observed, but their magnitude was weak, with absolute r values below 0.20. Therefore, these findings should not be interpreted as clinically meaningful in isolation.

The most frequent statistically significant correlations concerned cognitive impairment at ICU discharge. These associations were generally inverse, particularly for CRP and selected PCT measurements, indicating lower cognitive domain scores among patients with higher inflammatory marker concentrations. This direction was not consistent with a simple hypothesis that higher inflammation uniformly predicts worse cognitive outcomes. These findings may reflect confounding, survivor selection, timing of assessment, admission diagnosis or multiple testing, and should be interpreted as exploratory.

A weak positive correlation was observed between IL-6 measured within the first 12 h and psychological impairment at ICU discharge. However, this association was not consistently reproduced across all follow-up time points. Overall, the correlation matrix suggests that inflammatory biomarkers may be related to selected PICSQ domains but does not support a robust or uniform association with global PICSQ severity. To assess threshold associations between inflammatory markers and PICSQ symptom severity, mean PICSQ scores were compared across biomarker categories. These analyses were exploratory in nature and interpreted with respect to subgroup size, effect size, and consistency across assessment time points. A *p*-value below 0.05 was considered statistically significant for descriptive purposes only, without formal multiplicity adjustment.

This analysis did not demonstrate a consistent association between CRP level category and overall PICSQ symptom severity. Mean PICSQ scores were comparable across the assessment time points for patients with CRP values 5 mg/L or less and those with CRP values above 5 mg/L.

For PCT, a selected, unadjusted threshold association was observed for PCT ≥ 2 ng/mL on day 4 and overall PICSQ symptom severity at 3 months. However, because this was an exploratory comparison and the subgroup sizes were limited, this result should be interpreted cautiously and in conjunction with the adjusted models.

No consistent threshold-based association was observed between IL-6 concentration category and PICSQ total symptom severity after intensive care unit discharge, at 2 months, or at 3 months (Table 4).

Analyses stratified by PCT and interleukin-6 concentration categories are presented in Table 5 and Table 6. Overall, these threshold-based comparisons did not demonstrate a consistent association between inflammatory biomarker category and total PICSQ severity across all assessment time points.

For PCT, total PICSQ scores were generally comparable across concentration categories at ICU discharge and at 2 months after discharge. A selected unadjusted association was observed for PCT ≥ 2 ng/mL on day 4 with higher total PICSQ severity at 3 months. However, this finding was based on a limited subgroup with elevated PCT, was not adjusted for multiple comparisons, and should therefore be interpreted as an exploratory descriptive signal rather than as evidence of independent predictive value.

For interleukin-6, comparisons based on the exploratory threshold of >10 pg/mL did not show a consistent association with total PICSQ severity at ICU discharge, 2 months, or 3 months after discharge. These results should be interpreted together with the domain-specific and adjusted analyses presented in the Supplementary Materials, particularly because selected psychological domain associations were observed in unadjusted analyses, whereas fully adjusted linear regression models did not confirm statistically significant independent associations at *p* < 0.05.

The study conducted a comparative analysis of mean psychological domain scores across groups stratified by concentrations of selected inflammatory markers. These analyses were exploratory and should be interpreted in relation to subgroup size, effect size and consistency across time points.

CRP elevation after one week of hospitalization was associated with higher psychological domain scores at 3 months in an unadjusted threshold-based comparison. No consistent association was observed at other CRP time points. Detailed data are presented in Supplementary Table S1.

For PCT, higher psychological domain scores at 3 months were observed among patients with elevated PCT on day 2 and day 4, with the day 4 threshold of ≥2 ng/mL showing the most consistent signal in unadjusted comparisons. Detailed data are presented in Supplementary Table S2.

For IL-6, higher psychological domain scores at 3 months were observed among patients with IL-6 > 10 pg/mL on days 2 and 4. Detailed data are presented in Supplementary Table S3.

Cognitive domain analyses showed selected statistically significant associations for CRP and IL-6; however, the direction of these associations was not consistent with a simple hypothesis that higher inflammatory biomarker concentrations are associated with worse cognitive outcomes. In CRP-based comparisons, elevated CRP at 12 h, day 2, and day 4 was associated with lower cognitive domain scores at ICU discharge. Similar inverse or borderline inverse differences were observed in selected 3-month comparisons. Detailed data are presented in Supplementary Table S4.

PCT concentration categories were not consistently associated with cognitive domain scores at ICU discharge, 2 months or 3 months. Detailed data are presented in Supplementary Table S5.

For IL-6, significant differences were observed, including lower cognitive domain scores among patients with IL-6 > 10 pg/mL on day 2 and day 4 at 3 months, and among patients with elevated IL-6 after 2 weeks at ICU discharge. These findings should be interpreted cautiously because they were weak, inconsistent across time points and opposite to a simple directional inflammatory hypothesis. Detailed data are presented in Supplementary Table S6.

Physical domain analyses did not show a consistent association between CRP, PCT, or IL-6 concentration categories and the severity of physical impairment at ICU discharge, 2 months, or 3 months. Detailed data are presented in Supplementary Tables S7–S9.

Selected unadjusted associations between inflammatory markers and PICSQ severity at 3 months are summarized in Supplementary Table S10. The most consistent exploratory signal concerned the psychological domain. CRP > 5 mg/L after one week, PCT ≥ 2 ng/mL on day 4, IL-6 > 10 pg/mL on day 2, and IL 6 > 10 pg/mL on day 4 were associated with higher psychological domain scores at 3 months in unadjusted comparisons. PCT ≥ 2 ng/mL on day 4 was also associated with a higher total PICSQ score at 3 months in an unadjusted comparison. The observed effect sizes ranged from small to moderate. Because these analyses were exploratory and not corrected for multiple comparisons, their clinical relevance should be interpreted with caution.

Logistic regression was used to explore the risk of more severe psychological impairment at 3 months, defined as a PICSQ psychological domain score ≥ 4. In unadjusted analyses, CRP > 5 mg/L after 1 week, PCT ≥ 2 ng/mL on day 4, IL-6 > 10 pg/mL on day 2, and IL-6 > 10 pg/mL on day 4 were associated with higher odds of more severe psychological impairment. After adjustment for age, sex, reason for ICU admission, and length of hospitalization, only IL-6 > 10 pg/mL on day 2 remained statistically significant. Full model specifications, subgroup denominators and confidence intervals are presented in Supplementary Table S11.

In fully adjusted linear regression models including age, sex, reason for ICU admission and length of hospitalization, none of the selected biomarker thresholds remained statistically significant at *p* < 0.05. The strongest borderline associations were observed for PCT ≥ 2 ng/mL on day 4 in relation to total PICSQ score at 3 months (β = 0.44, 95% CI minus 0.07 to 0.94, *p* = 0.089) and IL-6 > 10 pg/mL on day 2 in relation to psychological domain severity at 3 months (β = 0.46, 95% CI minus 0.005 to 0.92, *p* = 0.052). Detailed coefficients, confidence intervals, *p* values, adjusted R^2^ values and covariates are presented in Supplementary Table S12.

## 5. Discussion

This prospective cohort study evaluated whether serial inflammatory biomarker concentrations measured during ICU hospitalization were associated with subsequent PICSQ severity. The main finding was that CRP, PCT, and IL-6 were not consistently associated with global PICSQ severity across all assessment time points. Selected exploratory associations were observed, particularly in the psychological domain at the 3-month follow-up. In adjusted logistic regression, IL-6 > 10 pg/mL on day 2 was associated with higher odds of more severe psychological impairment at 3 months. In fully adjusted linear regression models, the selected associations involving PCT and IL-6 were directionally consistent but did not reach statistical significance at *p* < 0.05. Correlation analyses also showed several weak inverse associations between inflammatory markers and cognitive scores, underscoring that the relationship between inflammation and PICS domains is unlikely to be linear or uniform.

The divergent cognitive findings require caution. Although inflammatory pathways may contribute to cognitive dysfunction after critical illness, several observed cognitive associations were inverse and did not support a simple directional relationship. Possible explanations include confounding by ICU admission diagnosis, differences in sedation and delirium trajectories, survivor bias, timing of assessment, subgroup size limitations, and multiple testing. These findings should be regarded as hypothesis-generating rather than clinically actionable.

Current literature indicates that PICS is a multidimensional phenomenon encompassing persistent cognitive impairment, reduced physical function, and psychological disturbances developing after critical illness. It is increasingly emphasized that the chronic sequelae of intensive care result not only from the severity of the acute illness episode but also from persistent immune dysregulation, neuroinflammation, endothelial dysfunction, and metabolic disturbances persisting after hospital discharge [18]. A systematic review published in 2024 demonstrated that among the biomarkers analyzed, CRP, IL-8, and IL-10 were most frequently associated with poorer outcomes following intensive care, thereby supporting the biological plausibility of inflammatory activation in the pathogenesis of long-term complications [18]. The present results are consistent with this concept only in part, because the observed associations were domain-specific, generally weak, and not uniformly reproduced across analytical approaches.

Our study did not demonstrate a consistent association between elevated CRP levels and overall PICSQ severity. Some CRP-related findings were observed in domain-specific analyses, but they were not uniform across assessment time points and included weak inverse associations with cognitive domain scores. Therefore, CRP should not be interpreted as a standalone marker of worse cognitive or psychological outcomes in this cohort. This is important because CRP serves as a secondary marker of IL-6-mediated hepatic acute phase activation and reflects the intensity of systemic inflammation, but it is not specific to neuroinflammatory injury. In the general population, higher CRP concentrations have been linked to depression, poorer cognitive functioning, and an increased risk of neurodegeneration. In patients recovering from severe illness, CRP may indicate prolonged biological stress, catabolism, or impaired recovery; however, our findings suggest that its clinical utility for PICS risk assessment is limited when used in isolation [19,20,21,22].

Interleukin-6 remains biologically plausible as a marker related to later psychological symptoms after critical illness. IL-6 is a central cytokine of the acute phase response and is involved in the activation of innate immunity, the regulation of the hypothalamic–pituitary–adrenal axis, blood–brain barrier permeability, microglial activation, and hippocampal neuroplasticity. Elevated IL-6 concentrations are observed in patients with sepsis, ARDS, polytrauma, and delirium. In studies of postoperative delirium, IL-6 has been among the most frequently identified risk biomarkers, and a 2025 meta-analysis reported higher concentrations of IL-6 and CRP in patients who developed postoperative delirium [23,24]. In the present study, IL-6 > 10 pg/mL on day 2 showed the most consistent signal for the psychological domain, including a significant association in adjusted logistic regression for a psychological domain score ≥ 4 at 3 months. Nevertheless, in fully adjusted linear regression, the association with psychological domain severity was borderline and did not reach *p* < 0.05. This distinction is important and supports a cautious interpretation.

Procalcitonin also showed selected exploratory associations, particularly when measured on day 4 of hospitalization. In unadjusted threshold-based analyses, PCT ≥ 2 ng/mL on day 4 was associated with higher total PICSQ and psychological domain scores at 3 months. However, this association did not remain statistically significant in fully adjusted linear models or adjusted logistic regression. PCT is more strongly associated with bacterial infection than CRP, although it may also increase in severe systemic stress. Higher PCT values may therefore act as a proxy for infection burden, treatment intensity, prolonged mechanical ventilation, deeper sedation, longer hospitalization, or other features of more severe critical illness, each of which may contribute to subsequent PICS risk [25,26]. These findings suggest that PCT should be interpreted as a contextual marker rather than as an independent predictor of PICS severity.

The absence of a consistent association between inflammatory biomarkers and the physical domain of PICS is also clinically plausible. Physical impairment after ICU treatment is strongly influenced by immobilization, duration of mechanical ventilation, critical illness neuropathy and myopathy, nutritional status, sarcopenia, and access to early mobilization and rehabilitation. Inflammation may contribute to muscle catabolism and impaired regeneration, but CRP, PCT, and IL-6 may not be sufficiently specific or sensitive to capture the complex mechanisms underlying post ICU physical disability.

A strength of the present study was the serial assessment of inflammatory biomarkers rather than reliance on a single admission measurement. The results suggest that the timing and trajectory of inflammatory activation may be more informative than an isolated baseline value. This observation is consistent with clinical data indicating that persistently elevated inflammatory markers after an acute illness episode may be associated with poorer recovery and chronic inflammatory activation [27]. However, in the present cohort, time-dependent associations were not uniform across biomarkers, PICSQ domains, and follow-up time points. Therefore, the results should be interpreted as hypothesis-generating rather than confirmatory.

Overall, the present findings suggest that inflammatory biomarkers may be associated with selected PICSQ domains, particularly psychological symptoms, but they do not support a simple model in which higher inflammatory biomarker concentrations uniformly predict worse global PICS severity. The small magnitude of several statistically significant correlations, the presence of inverse cognitive associations, the lack of multiplicity correction, and the attenuation of selected findings in fully adjusted linear models all limit immediate clinical interpretation. CRP, PCT, and IL-6 should therefore not be used as standalone predictors of PICS. At most, they may contribute to broader risk models that also incorporate illness severity, delirium, sedation exposure, frailty, comorbidities, rehabilitation exposure, and post-discharge care pathways.

## 6. Clinical Implications

The obtained results may have practical relevance for the organization of post-ICU follow-up, but they should not be interpreted as sufficient evidence for biomarker-based risk stratification. Patients with elevated IL-6 or PCT during the early course of ICU hospitalization may represent a subgroup warranting closer clinical observation, particularly regarding psychological symptoms after discharge. However, such decisions should be based on a broader clinical risk profile rather than on inflammatory biomarkers alone.

In practical terms, inflammatory biomarkers may support broader clinical assessment of patients who could benefit from earlier psychological screening, cognitive assessment, rehabilitation planning, and structured outpatient follow-up after ICU discharge. Their potential role should be understood as supportive and exploratory rather than diagnostic or predictive. Before CRP, PCT, or IL-6 can be incorporated into routine PICS risk stratification pathways, the present findings require validation in larger multicenter cohorts and in models that include established clinical predictors of post-ICU recovery.

## 7. Study Limitations

Several limitations should be acknowledged. First, this was a single-center observational study, and causal inference cannot be made. The observed associations may reflect systemic inflammation, severity of critical illness, admission diagnosis, treatment intensity, sedation exposure, delirium, rehabilitation access or other unmeasured factors rather than a direct biological effect of inflammatory biomarkers on PICS.

Second, numerous comparisons were conducted across biomarkers, time points, PICSQ domains, and follow-up assessments. No formal correction for multiplicity was applied. This increases the risk of Type I errors and false-positive findings. Therefore, secondary and exploratory *p*-values should be interpreted descriptively, especially when close to 0.05.

Third, the study used an available prospective cohort without an a priori sample size calculation. With 267 complete observations, the study was adequately powered only for small-to-moderate single associations and was not powered for extensive subgroup analyses after accounting for multiple comparisons. Some biomarker-stratified subgroups were small or empty, reducing precision and potentially explaining the instability of estimates in threshold-based tables.

Fourth, the complete case design may have introduced selection bias. Patients without complete biomarker data, incomplete follow-up data, or who were unable to complete the questionnaire were not included in the corresponding analyses. The number of observations excluded from each analysis and the subgroup denominators are reported in the main and Supplementary Tables.

Fifth, threshold-based analyses used clinically plausible but heterogeneous cutoffs for CRP, PCT, and IL-6. All thresholds should therefore be interpreted as exploratory and require validation. Finally, follow-up was limited to 3 months, whereas PICS may persist for many months or years after critical illness.

## 8. Directions for Future Research

Future studies should include multicenter validation cohorts and multivariate models integrating inflammatory biomarkers with clinical parameters. Combining CRP, IL-6, and PCT with neuronal biomarkers—such as neurofilament light chain (NfL), GFAP, or tau—appears particularly promising. This would allow for increased accuracy in predicting cognitive impairment following ICU care.

## 9. Conclusions

In this prospective single-center cohort, serial CRP, PCT, and IL-6 concentrations were not consistently associated with total PICSQ severity across all assessment time points. Selected exploratory associations were observed at 3 months, particularly in the psychological domain. IL-6 > 10 pg/mL on day 2 remained associated with higher odds of more severe psychological impairment in adjusted logistic regression, with an adjusted OR of 3.61 (95% CI, 1.26 to 10.37; *p* = 0.017). In fully adjusted linear regression, PCT ≥ 2 ng/mL on day 4 showed a borderline association with total PICSQ score at 3 months, with β = 0.44, 95% CI minus 0.07 to 0.94, *p* = 0.089, and IL 6 > 10 pg/mL on day 2 showed a borderline association with psychological domain severity at 3 months, with β = 0.46, 95% CI minus 0.005 to 0.92, *p* = 0.052. Cognitive domain associations were weak, inconsistent, and in several analyses inverse, while no consistent association was observed for the physical domain. These findings suggest that inflammatory biomarkers may be associated with selected PICS domains, particularly psychological symptoms, but they do not establish causality or predictive performance. External validation and models incorporating clinical severity, delirium, sedation, frailty, rehabilitation, and follow-up variables are required before these biomarkers can be considered for PICS risk stratification. 

## Figures and Tables

**Table 1:** Demographic characteristics of the study cohort.

Indicator	Group	N	%	x ± SD (Min–Max) ME	
Age [year]	Total	267	100.00%	52.61 ± 13.94 (18–78) ME = 56	
	30 years and less	25	9.36%		
	31–40 years	14	5.24%		
	41–50 years	68	25.47%		
	51–60 years	55	20.60%		
	61–70 years	90	33.71%		
	71 years and above	15	5.62%		
Sex	Female	110	41.20%		
	Male	157	58.80%		
Time to hospitalization [day]	Total	267	100.00%	19.89 ± 9.32 (3–45) ME = 21	
	Hypothermia	19	7.12%	5.95 ± 2.70 (3–12) ME = 5	
	Gastrointestinal Bleeding	34	12.73%	11.59 ± 6.42 (4–24) ME = 9	
	Circulatory Failure	93	34.83%	25.27 ± 6.68 (9–45) ME = 25	
	Respiratory Failure	43	16.10%	24.95 ± 6.98 (11–37) ME = 26	
	Acute Renal Failure	43	16.10%	13.65 ± 6.07 (6–31) ME = 12	
	Acute Pancreatitis	11	4.12%	19.27 ± 11.47 (8–45) ME = 17	
	Multi-organ injury	24	8.99%	24.29 ± 5.38 (17–35) ME = 22	
Death	No	255	95.51%		
	Yes	12	4.49%		

N—sample size; SD—standard deviation; Min—minimum; Max—maximum; ME—median; x—mean. NOTE: The high completeness and accuracy of mortality data in the analyzed cohort resulted from both the eligibility criteria applied and the prospective patient follow-up model. Participation in the study was restricted exclusively to conscious individuals capable of cooperation and independent completion of research instruments. This entailed the exclusion of patients in the most critical clinical condition, including those under deep sedation or presenting with significant disturbances of consciousness. Consequently, the analyzed group comprised individuals with a relatively more favorable prognosis, which may have contributed to the observed low mortality rate. Concurrently, the implementation of structured organizational procedures facilitated achieving a high level of follow-up completeness. As early as discharge from the Intensive Care Unit, follow-up visit dates were scheduled, and patients were provided with detailed information about the subsequent stages of the study. In instances where an in-person visit was not feasible, certain assessments were conducted via a standardized telephone interview. This approach effectively minimized data loss during the long-term follow-up period and ensured the high quality and reliability of the analyzed endpoints.

**Table 2:** Descriptive statistics for the mean PICS score and the PICS scale on individual days of hospitalization.

PICS	N	x	−95%	+95%	ME	Min	Max	Q1	Q3	SD	Skewness	Kurtosis
PICS at discharge	267	12.34	12.09	12.59	12.00	8.00	17.00	11.00	14.00	2.09	−0.17	−0.68
Mental status at discharge	267	3.69	3.54	3.85	4.00	2.00	7.00	3.00	4.00	1.28	0.65	0.15
Cognitive assessment at discharge	267	5.78	5.64	5.92	6.00	3.00	8.00	5.00	7.00	1.14	−0.01	−0.52
Physical condition at discharge	267	3.44	3.28	3.60	3.00	1.00	6.00	3.00	4.00	1.34	−0.02	−0.47
PICS: 2 months post-discharge	267	12.27	12.08	12.46	12.00	8.00	16.00	11.00	13.00	1.59	−0.04	0.24
Mental status: 2 months post-discharge	267	3.57	3.44	3.70	4.00	2.00	7.00	3.00	4.00	1.05	0.23	−0.01
Cognitive assessment: 2 months post-discharge	267	5.76	5.64	5.88	6.00	4.00	8.00	5.00	6.00	1.00	0.45	−0.49
Physical therapy: 2 months from discharge	267	3.46	3.33	3.58	3.00	1.00	6.00	3.00	4.00	1.05	0.35	0.14
PICS 3 months post-discharge	267	11.48	11.36	11.61	12.00	8.00	15.00	11.00	12.00	1.06	−0.29	2.30
Mental status: 3 months post-discharge	267	3.09	2.97	3.21	3.00	2.00	6.00	2.00	4.00	1.03	0.09	−1.51
Cognitive assessment 3 months post-discharge	267	5.50	5.43	5.58	5.00	4.00	7.00	5.00	6.00	0.63	0.35	−0.25
Physical examination: 3 months post-discharge	267	3.06	2.98	3.14	3.00	1.00	6.00	3.00	3.00	0.63	0.79	0.12

PICS—Post-Intensive Care Syndrome; N—number; ME—median; Min—minimum; Max—maximum; SD—standard deviation; x—mean; Q1—first quartile; Q3—third quartile.

**Table 3:** Pearson’s R correlations between selected inflammatory parameters examined and the development of Post-Intensive Care Syndrome disorders.

Pearson R Correlations	PICS at Discharge	Mental Status at Discharge	Cognitive Assessment at Discharge	Physical Examination upon Discharge	PICS: 2 Months Post-Discharge	Mental status: 2 Months Post-Discharge	Cognitive Assessment: 2 Months Post-Discharge	Physical Therapy: 2 Months from Discharge	PICS: 3 Months Post-Discharge	Mental Status: 3 Months Post-Discharge	Cognitive Assessment 3 Months Post-Discharge	Physical Examination: 3 Months Post-Discharge
CRP 12 h after admission [mg/L]	0.028 (0.656)	0.032 (0.613)	−0.159 * (0.011)	0.138 * (0.027)	0.051 (0.412)	0.009 (0.889)	0.014 (0.823)	0.067 (0.286)	0.085 (0.174)	0.082 (0.190)	−0.057 (0.361)	0.074 (0.235)
CRP 2nd day after admission [mg/L]	0.022 (0.731)	−0.013 (0.840)	−0.127 * (0.043)	0.121 (0.054)	0.053 (0.399)	−0.020 (0.746)	0.005 (0.933)	0.112 (0.075)	0.076 (0.225)	0.068 (0.282)	−0.026 (0.682)	0.039 (0.538)
CRP 4 days after admission [mg/L]	0.014 (0.818)	−0.004 (0.951)	−0.145 * (0.020)	0.111 (0.076)	0.066 (0.291)	−0.002 (0.975)	0.037 (0.554)	0.087 (0.163)	0.091 (0.145)	0.099 (0.114)	−0.050 (0.423)	0.021 (0.736)
CRP 1 week after admission [mg/L]	0.003 (0.959)	0.004 (0.953)	−0.169 * (0.007)	0.098 (0.116)	0.048 (0.446)	0.005 (0.931)	0.015 (0.807)	0.059 (0.349)	0.079 (0.207)	0.075 (0.229)	−0.024 (0.700)	0.001 (0.984)
CRP 2 weeks after admission [mg/L]	−0.010 (0.874)	−0.031 (0.617)	−0.185 * (0.003)	0.121 (0.053)	0.036 (0.561)	−0.027 (0.665)	0.004 (0.945)	0.075 (0.230)	0.073 (0.243)	0.062 (0.319)	−0.003 (0.963)	−0.007 (0.910)
PCT 12 h after admiddion [ng/mL]	−0.043 (0.494)	−0.072 (0.252)	−0.052 (0.410)	−0.012 (0.843)	0.087 (0.165)	0.009 (0.888)	0.029 (0.650)	0.109 (0.081)	0.092 (0.141)	0.106 (0.090)	−0.032 (0.615)	0.048 (0.447)
PCT 2nd day after admission [ng/mL]	0.032 (0.611)	−0.002 (0.975)	−0.097 (0.123)	0.108 (0.084)	0.061 (0.330)	0.037 (0.555)	0.060 (0.342)	0.046 (0.461)	0.087 (0.165)	0.058 (0.356)	−0.011 (0.857)	0.049 (0.432)
PCT 4 days after admission [ng/mL]	0.024 (0.708)	−0.008 (0.905)	−0.116 (0.064)	0.115 (0.066)	0.044 (0.483)	0.023 (0.718)	0.036 (0.567)	0.037 (0.560)	0.105 (0.095)	0.093 (0.138)	−0.024 (0.700)	0.042 (0.507)
PCT 1 week after admission [ng/mL]	−0.015 (0.814)	−0.017 (0.783)	−0.142 * (0.023)	0.080 (0.200)	0.028 (0.656)	−0.005 (0.941)	0.039 (0.534)	0.028 (0.653)	0.093 (0.136)	0.071 (0.260)	−0.010 (0.878)	0.039 (0.539)
PCT 2 weeks after admission [ng/mL]	−0.022 (0.722)	−0.027 (0.665)	−0.146 * (0.019)	0.078 (0.212)	0.018 (0.769)	−0.017 (0.789)	0.019 (0.757)	0.033 (0.603)	0.100 (0.112)	0.082 (0.189)	−0.017 (0.787)	0.040 (0.520)
IL-6 h after admission [pg/mL]	0.096 (0.127)	0.144 * (0.021)	−0.012 (0.853)	0.082 (0.193)	−0.020 (0.753)	0.035 (0.581)	−0.046 (0.462)	0.029 (0.645)	0.044 (0.482)	0.082 (0.188)	−0.074 (0.240)	−0.010 (0.879)
IL-6 2nd day after admission [pg/mL]	0.065 (0.301)	0.102 (0.104)	−0.056 (0.372)	0.086 (0.171)	−0.001 (0.991)	0.049 (0.439)	−0.033 (0.600)	0.042 (0.505)	0.085 (0.175)	0.115 (0.066)	−0.070 (0.266)	0.010 (0.876)
IL-6 4 days after admission [pg/mL]	−0.023 (0.718)	−0.018 (0.777)	−0.119 (0.056)	0.063 (0.315)	−0.016 (0.800)	0.011 (0.866)	−0.050 (0.428)	0.035 (0.579)	0.065 (0.303)	0.068 (0.279)	−0.046 (0.463)	0.005 (0.934)
IL-6 1 week after admission [pg/mL]	−0.076 (0.228)	−0.078 (0.213)	−0.137 * (0.028)	0.021 (0.739)	−0.026 (0.674)	−0.021 (0.738)	−0.022 (0.725)	0.010 (0.871)	0.038 (0.545)	0.013 (0.842)	−0.003 (0.958)	0.002 (0.969)
IL-6 2 weeks after admission [pg/mL]	−0.074 (0.240)	−0.117 (0.061)	−0.143 * (0.022)	0.054 (0.389)	0.010 (0.879)	−0.044 (0.483)	0.022 (0.724)	0.063 (0.318)	0.037 (0.552)	0.006 (0.927)	0.008 (0.897)	0.003 (0.964)

PICS—Post-Intensive Care Syndrome; CRP—C-reactive protein; PCT—procalcitonin; IL-6—interleukin-6; mg/L—milligrams per liter; pg/mL—picograms per milliliter. Values are Pearson r coefficients with unadjusted *p* values in parentheses. The effective sample size for the correlation matrix was N = 256 with a complete-case analysis. * *p* < 0.05, uncorrected. Because no formal correction for multiple comparisons was applied, *p*-values should be interpreted descriptively.

**Table 4:** Significance of the severity of Post-Intensive Care Syndrome symptoms in relation to C-reactive protein concentration levels.

	PICS at Discharge				*p*	PICS: 2 Months Post-Discharge				*p*	PICS 3 Months Post-Discharge				*p*
	x	SD	x	SD		x	SD	x	SD		x	SD	x	SD	
	Norm		Above 5 mg/L			Norm		Above 5 mg/L			Norm		Above 5 mg/L		
CRP 12 h after admission [mg/L]	12.37	2.10	12.19	2.03	0.61	12.30	1.65	12.14	1.25	0.55	11.46	1.10	11.63	0.82	0.33
CRP 2nd day after admission [mg/L]	12.37	2.10	12.19	2.03	0.61	12.30	1.65	12.14	1.25	0.55	11.46	1.10	11.63	0.82	0.33
CRP 4 days after admission [mg/L]	12.37	2.10	12.20	2.08	0.63	12.30	1.65	12.10	1.24	0.46	11.46	1.10	11.61	0.83	0.39
CRP 1 week after admission [mg/L]	12.35	2.09	12.26	2.03	0.82	12.27	1.64	12.19	1.08	0.79	11.46	1.11	11.74	0.77	0.18
CRP 2 weeks after admission [mg/L]	12.35	2.05	12.35	1.96	1.00	12.27	1.58	12.31	0.93	0.92	11.51	1.15	11.85	0.61	0.14

PICS—Post-Intensive Care Syndrome; CRP—C-reactive protein; SD—standard deviation; *p*—significance level; x—mean; mg/L—milligrams per liter.

**Table 5:** Significance of the severity of Post-Intensive Care Syndrome disorders in relation to procalcitonin concentration levels.

	PICS Related Impairments at Discharge						*p*	PICS Symptoms 2 Months Post-Discharge						*p*	PICS Symptoms 3 Months Post-Discharge						*p*
	x	SD	x	SD	x	SD		x	SD	x	SD	x	SD		x	SD	x	SD	x	SD	
	PCT 0.5 ng/mL or Lower		PCT Above 0.5 and Below 2 ng/mL		PCT 2 ng/mL or Higher			PCT 0.5 ng/mL or Lower		PCT Above 0.5 and Below 2 ng/mL		PCT 2 ng/mL or Higher			PCT 0.5 ng/mL or Lower		PCT Above 0.5 and Below 2 ng/mL		PCT 2 ng/mL or Higher		
PCT 12 h after admiddion [ng/mL]	12.34	2.10	11.67	0.58	NA	NA	0.58	12.26	1.59	13.33	1.53	NA	NA	0.25	11.47	1.06	12.33	0.58	NA	NA	0.16
PCT 2nd day after admission [ng/mL]]	12.31	2.12	12.67	2.31	12.61	1.69	0.81	12.26	1.63	11.67	0.58	12.61	0.98	0.53	11.45	1.09	12.33	0.58	11.83	0.62	0.13
PCT 4 days after admission [ng/mL]	12.32	2.12	NA	NA	12.50	1.77	0.70	12.26	1.63	NA	NA	12.45	0.96	0.58	11.44	1.09	NA	NA	11.91	0.61	0.05
PCT 1 week after admission [ng/mL]	12.34	2.10	11.00	NA	12.36	1.99	0.81	12.26	1.63	12.00	NA	12.41	1.01	0.90	11.45	1.09	12.00	NA	11.86	0.64	0.19
PCT 2 weeks after admission [ng/mL]	12.34	2.10	14.00	NA	12.17	1.95	0.69	12.26	1.62	14.00	NA	12.33	1.03	0.55	11.46	1.08	11.00	NA	11.89	0.68	0.22

PICS—Post-Intensive Care Syndrome; SD—standard deviation; *p*—significance level; x—mean; ng/mL—nanograms per milliliter; NA—indicates that no patients were present in the corresponding PCT category at this time point; therefore, mean and/or SD could not be calculated. NA indicates that no patients were present in the corresponding PCT category or that SD could not be calculated because the subgroup included only one patient. *p*-values were calculated using available non-empty groups and should be interpreted descriptively.

**Table 6:** Significance of the severity of Post-Intensive Care Syndrome symptoms in relation to interleukin-6 concentration levels.

	PICS Related Impairments at Discharge				*p*	PICS Symptoms 2 Months Post-Discharge				*p*	PICS Symptoms 3 Months Post-Discharge				*p*
	x	SD	x	SD		x	SD	x	SD		x	SD	x	SD	
	Norm Level		Acute Infection			Norm Level		Acute Infection			Norm Level		Acyte Infection		
IL-6 h after admission [pg/mL]	12.34	2.08	12.27	2.45	0.92	12.28	1.62	12.09	0.54	0.70	11.46	1.08	11.91	0.54	0.18
IL-6 2nd day after admission [pg/mL]	12.34	2.09	12.36	2.14	0.96	12.28	1.64	12.20	1.00	0.81	11.45	1.09	11.76	0.78	0.17
IL-6 4 days after admission [pg/mL]	12.32	2.11	12.54	2.00	0.61	12.27	1.64	12.35	1.09	0.81	11.45	1.09	11.73	0.78	0.21
IL-6 1 week after admission [pg/mL]	12.33	2.11	12.33	1.98	0.99	12.27	1.64	12.30	1.10	0.95	11.46	1.11	11.70	0.78	0.27
IL-6 2 weeks after admission [pg/mL]	12.40	2.07	12.05	1.84	0.49	12.30	1.58	12.21	0.92	0.81	11.51	1.14	11.79	0.63	0.30

PICS—Post-Intensive Care Syndrome; SD—standard deviation; *p*—significance level; x—mean; pg/mL—picograms per milliliter.

## Data Availability

The datasets used and analyzed in this study are available from the corresponding author upon reasonable request.

## Supplementary Materials

The following supporting information can be downloaded at https://www.mdpi.com/article/10.3390/diagnostics16152361/s1, Table S1. Association between CRP concentration category and psychological PICSQ domain scores. Table S2. Association between PCT concentration category and psychological PICSQ domain scores. Table S3. Association between IL 6 concentration category and psychological PICSQ domain scores. Table S4. Association between CRP concentration category and cognitive PICSQ domain scores. Table S5. Association between PCT concentration category and cognitive PICSQ domain scores. Table S6. Association between IL-6 concentration category and cognitive PICSQ domain scores. Table S7. Association between CRP concentration category and physical PICSQ domain scores. Table S8. Association between PCT concentration category and physical PICSQ domain scores. Table S9. Association between IL-6 concentration category and physical PICSQ domain scores. Table S10. Selected unadjusted associations between inflammatory markers and PICSQ severity at 3 months. Table S11. Logistic regression models for higher psychological PICSQ domain severity at 3 months. Table S12. Multivariable linear regression models for selected PICSQ endpoints at 3 months. These tables include domain-specific group comparisons, selected unadjusted associations, logistic regression models, and multivariable linear regression models. Effective sample sizes, subgroup denominators, effect estimates, confidence intervals, and *p*-values are reported where applicable.

## Abbreviations

ARDS	Acute Respiratory Distress Syndrome
CAM-ICU	Confusion Assessment Method for the Intensive Care Unit
CRP	C-reactive protein
ELISA	Enzyme-Linked Immunosorbent Assay
ICU	Intensive Care Unit
IL-6	Interleukin 6
IL-8	Interleukin 8
IL-10	Interleukin 10
NfL	Neurofilament Light Chain
PCT	Procalcitonin
PICS	Post-Intensive Care Syndrome
PICSQ	Post-Intensive Care Syndrome Questionnaire
SD	Standard Deviation
GFAP	Glial Fibrillary Acidic Protein
ANOVA	Analysis of Variance
CI	Confidence Interval
Q1	First Quartile
Q3	Third Quartile
Min	Minimum
Max	Maximum
ME	Median
HPA axis	Hypothalamic–Pituitary–Adrenal Axis
