# Supplementary material for: Inflammatory Biomarkers and Post-Intensive Care Syndrome: A Prospective Cohort Study
Source: Diagnostics (Basel). 2026 Jul 27;16(15):2361. doi: 10.3390/diagnostics16152361 (PMC13465726; doi:10.3390/diagnostics16152361)
Supplement: Supplementary file 1 [file diagnostics-16-02361-s001.zip › diagnostics-4369119-supplementary.pdf]

SUPPLEMENTARY MATERIALS

Table S1. Association between CRP concentration category and psychological PICSQ domain scores.

CRP	N	CRP above 5 mg/l	Mental disorders on the day of discharge				p	Mental disorders 2 months after discharge				p	Mental disorders 3 months after discharge				p
			x	SD	x	SD		x	SD	x	SD		x	SD	x	SD	
			Norm		Above 5 mg/l			Norm		Above 5 mg/l			Norm		Above 5 mg/l		
CRP 12 hours after admission [mg/l]	224	43	3.69	1.24	3.72	1.45	0.88	3.58	1.07	3.51	0.96	0.70	3.04	1.03	3.35	0.95	0.07
CRP 2nd day after admission [mg/l]	224	43	3.69	1.24	3.72	1.45	0.88	3.58	1.07	3.51	0.96	0.70	3.04	1.03	3.35	0.95	0.07
CRP 4 days after admission [mg/l]	224	41	3.69	1.24	3.76	1.48	0.75	3.58	1.07	3.49	0.95	0.61	3.04	1.03	3.32	0.96	0.11
CRP 1 week after admission [mg/l]	216	31	3.68	1.22	3.68	1.40	1.00	3.57	1.07	3.65	0.88	0.72	3.06	1.03	3.45	0.93	0.04
CRP 2 weeks after admission [mg/l]	168	26	3.68	1.22	3.69	1.41	0.98	3.58	1.09	3.65	0.80	0.75	3.14	1.03	3.46	0.95	0.13

CRP – C-reactive protein; SD – standard deviation; p – significance level; x – mean; mg/L – milligrams per liter; N – number of patients

Data are presented as the mean and standard deviation. The effective sample size and subgroup denominators are reported for each biomarker time point. P values are descriptive because no formal correction for multiple comparisons was applied.

Table S2. Association between PCT concentration category and psychological PICSQ domain scores.

PCT	PCT 0.5 ng/mL or lower (number of patients)	PCT above 0.5 and below 2 ng/mL (number of patients)	PCT 2 ng/mL or higher (number of patients)	Mental disorders on the day of discharge						p	Mental disorders 2 months after discharge						p	Mental disorders 3 months after discharge						p
				x	SD	x	SD	x	SD		x	SD	x	SD	x	SD		x	SD	x	SD	x	SD	
				PCT 0.5 ng/mL or lower		PCT above 0.5 and below 2 ng/mL		PCT 2 ng/mL or higher			PCT 0.5 ng/mL or lower		PCT above 0.5 and below 2 ng/mL		PCT 2 ng/mL or higher			PCT 0.5 ng/mL or lower		PCT above 0.5 and below 2 ng/mL		PCT 2 ng/mL or higher		
PCT 12 hours after admission [ng/mL]	246	3	NA	3.70	1.28	3.00	1.00	NA		0.35	3.57	1.06	3.67	0.58	NA		0.87	3.08	1.03	4.00	0.00	NA		0.12
PCT 2nd day after admission [ng/mL]	246	3	18	3.69	1.27	4.67	1.15	3.56	1.34	0.38	3.55	1.07	3.00	1.00	3.89	0.76	0.27	3.05	1.03	4.33	0.58	3.39	0.92	0.04
PCT 4 days after admission [ng/mL]	245	NA	22	3.70	1.27	NA		3.64	1.36	0.83	3.55	1.07	NA		3.77	0.81	0.35	3.05	1.03	NA		3.55	0.91	0.03
PCT 1 week after admission [ng/mL]	244	1	22	3.70	1.27	2.00	NA	3.68	1.32	0.41	3.56	1.07	4.00	NA	3.68	0.89	0.80	3.05	1.03	4.00	NA	3.45	0.96	0.14
PCT 2 weeks after admission [ng/mL]	248	1	18	3.70	1.27	5.00		3.56	1.38	0.53	3.57	1.07	5.00		3.50	0.86	0.38	3.07	1.03	2.00		3.44	0.98	0.18

PCT – procalcitonin; SD – standard deviation; p – significance level; x – mean; ng/mL – nanograms per milliliter; NA - indicates that no patients were present in the corresponding PCT category at this time point; therefore, mean and/or SD could not be calculated.

Data are presented as the mean and standard deviation. The effective sample size and subgroup denominators are reported for each biomarker time point. P values are descriptive because no formal correction for multiple comparisons was applied.

Subgroup denominators are reported separately for each PCT measurement time point because patient allocation to PCT categories changed over time. NA indicates that no patients were present in the corresponding PCT category or that SD could not be calculated because the subgroup included only one patient. P values are exploratory and were calculated using the available non empty groups at each time point.

Table S3. Association between IL 6 concentration category and psychological PICSQ domain scores.

	Mental disorders on the day of discharge					Mental disorders 2 months after discharge					Mental disorders 3 months after discharge				
	x	SD	x	SD		x	SD	x	SD		x	SD	x	SD	
	IL-6 10 pg/mL or lower		IL-6 above 10 pg/mL		p	IL-6 10 pg/mL or lower		IL-6 above 10 pg/mL		p	IL-6 10 pg/mL or lower		IL-6 above 1 0 pg/mL		p
IL-6 hours after admission [pg/ml]	3.71	1.26	3.18	1.54	0.18	3.57	1.06	3.64	0.81	0.83	3.07	1.03	3.64	0.81	0.07
IL-6 2nd day after admission [pg/ml]	3.69	1.27	3.64	1.35	0.83	3.55	1.08	3.72	0.74	0.45	3.04	1.03	3.56	0.82	0.02
IL-6 4 days after admission [pg/ml]	3.69	1.27	3.69	1.38	0.99	3.54	1.08	3.85	0.73	0.16	3.04	1.04	3.46	0.86	0.05
IL-6 1 week after admission [pg/ml]	3.66	1.20	3.63	1.39	0.89	3.57	1.06	3.78	0.80	0.33	3.05	1.03	3.41	0.89	0.09
IL-6 2 weeks after admission [pg/ml]	3.72	1.26	3.53	1.17	0.51	3.60	1.08	3.53	0.84	0.76	3.14	1.02	3.37	0.96	0.35

IL-6 – interleukin-6; pg/ml – picograms per milliliter; SD – standard deviation; p – significance level; x – mean

Data are presented as the mean and standard deviation. The effective sample size and subgroup denominators are reported for each biomarker time point. P values are descriptive because no formal correction for multiple comparisons was applied.
The IL-6 threshold above 10 pg/mL was used for exploratory threshold-based analyses and should not be interpreted as a standalone diagnosis of acute infection.

Table S4. Association between CRP concentration category and cognitive PICSQ domain scores.

	N	CRP above 5 mg/l	Cognitive impairment on the day of discharge				p	Cognitive impairment 2 months post-discharge				p	Cognitive impairment 3 months after discharge				p
			x	SD	x	SD		x	SD	x	SD		x	SD	x	SD	
			Norm		Above 5 mg/l			Norm		Above 5 mg/l			Norm		Above 5 mg/l		
CRP 12 hours after admission [mg/l]	224	43	5.84	1.14	5.44	1.12	0.03	5.77	1.01	5.70	0.99	0.66	5.54	0.64	5.33	0.57	0.05
CRP 2nd day after admission [mg/l]	224	43	5.84	1.14	5.44	1.12	0.03	5.77	1.01	5.70	0.99	0.66	5.54	0.64	5.33	0.57	0.05
CRP 4 days after admission [mg/l]	224	41	5.84	1.14	5.44	1.14	0.04	5.77	1.01	5.66	0.94	0.50	5.54	0.64	5.34	0.57	0.07
CRP 1 week after admission [mg/l]	216	31	5.83	1.13	5.48	1.09	0.11	5.78	1.00	5.61	0.88	0.39	5.53	0.64	5.32	0.54	0.08
CRP 2 weeks after admission [mg/l]	168	26	5.85	1.12	5.42	0.90	0.07	5.79	0.99	5.65	0.89	0.52	5.50	0.66	5.38	0.50	0.39

CRP – C-reactive protein; SD – standard deviation; p – significance level; x – mean; mg/L – milligrams per liter

Data are presented as the mean and standard deviation. The effective sample size and subgroup denominators are reported for each biomarker time point. P values are descriptive because no formal correction for multiple comparisons was applied.

Table S5. Association between PCT concentration category and cognitive PICSQ domain scores.

	PCT 0.5 ng/mL or lower (number of patients)	PCT above 0.5 and below 2 ng/mL (number of patients)	PCT 2 ng/mL or higher (number of patients)	Cognitive impairment on the day of discharge						p	Cognitive impairment 2 months post-discharge						p	Cognitive impairment 3 months after discharge						p
				x	SD	x	SD	x	SD		x	SD	x	SD	x	SD		x	SD	x	SD	x	SD	
				PCT 0.5 ng/mL or lower		PCT above 0.5 and below 2 ng/mL		PCT 2 ng/mL or higher			PCT 0.5 ng/mL or lower		PCT above 0.5 and below 2 ng/mL		PCT 2 ng/mL or higher			PCT 0.5 ng/mL or lower		PCT above 0.5 and below 2 ng/mL		PCT 2 ng/mL or higher		
PCT 12 hours after admission [ng/mL]	246	3	NA	5.78	1.15	5.33	0.58			0.50	5.76	1.00	6.00	1.00			0.68	5.50	0.63	5.33	0.58			0.64
PCT 2nd day after admission [ng/mL]	246	3	18	5.78	1.16	6.00	1.00	5.67	1.03	0.87	5.76	1.01	5.33	0.58	5.89	0.96	0.66	5.51	0.64	5.33	0.58	5.39	0.50	0.66
PCT 4 days after admission [ng/mL]	245	NA	22	5.79	1.15	NA	NA	5.64	1.05	0.54	5.76	1.01	NA	NA	5.77	0.92	0.95	5.51	0.64	NA	NA	5.36	0.49	0.29
PCT 1 week after admission [ng/mL]	244	1	22	5.80	1.15	6.00	NA	5.55	1.10	0.60	5.76	1.01	5.00	NA	5.82	0.91	0.72	5.51	0.64	5.00	NA	5.41	0.50	0.56
PCT 2 weeks after admission [ng/mL]	248	1	18	5.81	1.15	6.00	NA	5.28	0.96	0.15	5.75	1.01	8.00	NA	5.78	0.81	0.08	5.50	0.64	6.00	NA	5.44	0.51	0.68

PCT – procalcitonin; SD – standard deviation; p – significance level; x – mean; ng/mL – nanogram per milliliter; NA - indicates that no patients were present in the corresponding PCT category at this time point; therefore, mean and/or SD could not be calculated.

Data are presented as the mean and standard deviation. The effective sample size and subgroup denominators are reported for each biomarker time point. P values are descriptive because no formal correction for multiple comparisons was applied.
Subgroup denominators are reported separately for each PCT measurement time point because patient allocation to PCT categories changed over time. NA indicates that no patients were present in the corresponding PCT category or that SD could not be calculated because the subgroup included only one patient. P values are exploratory and were calculated using the available non empty groups at each time point.

Table S6. Association between IL-6 concentration category and cognitive PICSQ domain scores.

	Cognitive impairment on the day of discharge				p	Cognitive impairment 2 months post-discharge				p	Cognitive impairment 3 months after discharge				p
	x	SD	x	SD		x	SD	x	SD		x	SD	x	SD	
	IL-6 10 pg/ml or lower		IL-6 above 10 pg/ml			IL-6 10 pg/ml or lower		IL-6 above 10 pg/ml			IL-6 10 pg/ml or lower		IL-6 above 10 pg/ml		
IL-6 hours after admission [pg/ml]	5.77	1.14	5.91	1.38	0.70	5.77	1.01	5.45	0.69	0.30	5.51	0.64	5.27	0.47	0.22
IL-6 2nd day after admission [pg/ml]	5.79	1.14	5.60	1.15	0.42	5.80	1.02	5.44	0.77	0.09	5.53	0.64	5.24	0.52	0.03
IL-6 4 days after admission [pg/ml]	5.79	1.15	5.69	1.09	0.69	5.78	1.01	5.62	0.94	0.42	5.53	0.64	5.27	0.53	0.05
IL-6 1 week after admission [pg/ml]	5.82	1.15	5.52	1.01	0.19	5.77	1.00	5.63	0.93	0.48	5.53	0.64	5.30	0.54	0.06
IL-6 2 weeks after admission [pg/ml]	5.84	1.11	5.21	0.92	0.02	5.79	1.01	5.63	0.76	0.50	5.50	0.66	5.42	0.51	0.63

IL-6 – interleukin-6; pg/ml – picograms per milliliter; SD – standard deviation; p – significance level; x – mean

Data are presented as the mean and standard deviation. The effective sample size and subgroup denominators are reported for each biomarker time point. P values are descriptive because no formal correction for multiple comparisons was applied.
The IL-6 threshold above 10 pg/mL was used for exploratory threshold-based analyses and should not be interpreted as a standalone diagnosis of acute infection.

Table S7. Association between CRP concentration category and physical PICSQ domain scores.

	N	CRP above 5 mg/l	Physical disturbances on the day of discharge				p	Physical disturbances 2 months after discharge				p	Physical disturbances months since discharge				p
			x	SD	x	SD		x	SD	x	SD		x	SD	x	SD	
			Norm		Above 5 mg/l			Norm		Above 5 mg/l			Norm		Above 5 mg/l		
CRP 12 hours after admission [mg/l]	224	43	3.42	1.36	3.51	1.24	0.70	3.46	1.08	3.47	0.91	0.96	3.07	0.66	3.02	0.41	0.68
CRP 2nd day after admission [mg/l]	224	43	3.42	1.36	3.51	1.24	0.70	3.46	1.08	3.47	0.91	0.96	3.07	0.66	3.02	0.41	0.68
CRP 4 days after admission [mg/l]	224	41	3.42	1.36	3.49	1.27	0.78	3.46	1.08	3.46	0.92	0.96	3.07	0.66	3.02	0.42	0.69
CRP 1 week after admission [mg/l]	216	31	3.42	1.37	3.71	1.27	0.27	3.44	1.08	3.48	0.93	0.81	3.06	0.67	3.06	0.44	1.00
CRP 2 weeks after admission [mg/l]	168	26	3.41	1.36	3.81	1.33	0.17	3.41	1.05	3.54	0.90	0.56	3.07	0.70	3.12	0.43	0.76

CRP – C-reactive protein; SD – standard deviation; p – significance level; x – mean; mg/L – milligrams per liter

Data are presented as the mean and standard deviation. The effective sample size and subgroup denominators are reported for each biomarker time point. P values are descriptive because no formal correction for multiple comparisons was applied.

Table S8. Association between PCT concentration category and physical PICSQ domain scores.

	PCT 0.5 ng/mL or lower (number of patients)	PCT above 0.5 and below 2 ng/mL (number of patients)	PCT 2 ng/mL or higher (number of patients)	Physical disturbances on the day of discharge						p	Physical disturbances 2 months after discharge						p	Physical disturbances 3 months after discharge						p
				x	SD	x	SD	x	SD		x	SD	x	SD	x	SD		x	SD	x	SD	x	SD	
				PCT 0.5 ng/mL or lower		PCT above 0.5 and below 2 ng/mL		PCT 2 ng/mL or higher			PCT 0.5 ng/mL or lower		PCT above 0.5 and below 2 ng/mL		PCT 2 ng/mL or higher			PCT 0.5 ng/mL or lower		PCT above 0.5 and below 2 ng/mL		PCT 2 ng/mL or higher		
PCT 12 hours after admission [ng/mL]	246	3	NA	3.44	1.34	3.33	0.58	NA		0.89	3.45	1.05	4.33	1.53	NA		0.15	3.06	0.63	3.33	0.58	NA		0.45
PCT 2nd day after admission [ng/mL	246	3	18	3.41	1.34	3.00	1.73	3.94	1.21	0.22	3.45	1.06	3.33	0.58	3.56	0.98	0.90	3.05	0.64	3.00	0.00	3.17	0.51	0.75
PCT 4 days after admission [µg/l]]	245	NA	22	3.40	1.34	NA		3.82	1.26	0.16	3.45	1.06	NA		3.50	0.91	0.84	3.05	0.64	Na		3.14	0.47	0.55
PCT 1 week after admission [ng/mL	244	1	22	3.41	1.34	3.00	NA	3.73	1.39	0.55	3.45	1.07	3.00	NA	3.50	0.91	0.89	3.05	0.64	3.00	NA	3.14	0.47	0.84
PCT 2 weeks after admission [ng/mL]	248	1	18	3.41	1.34	4.00	NA	3.78	1.26	0.49	3.45	1.06	3.00	NA	3.56	0.98	0.84	3.05	0.64	3.00	NA	3.17	0.51	0.76

PCT – procalcitonin; SD – standard deviation; p – significance level; x – mean; ng/mL – nanogramper mililiter; NA - indicates that no patients were present in the corresponding PCT category at this time point; therefore, mean and/or SD could not be calculated.

Data are presented as the mean and standard deviation. The effective sample size and subgroup denominators are reported for each biomarker time point. P values are descriptive because no formal correction for multiple comparisons was applied.
Subgroup denominators are reported separately for each PCT measurement time point because patient allocation to PCT categories changed over time. NA indicates that no patients were present in the corresponding PCT category or that SD could not be calculated because the subgroup included only one patient. P values are exploratory and were calculated using the available non empty groups at each time point.

Table S9. Association between IL-6 concentration category and physical PICSQ domain scores.

	Physical disturbances on the day of discharge				p	Physical disturbances 2 months after discharge				p	Physical disturbances 3 months after discharge				p
	x	SD	x	SD		x	SD	x	SD		x	SD	x	SD	
	IL-6 10 pg/ml or lower		IL-6 above 10 pg/ml			IL-6 10 pg/ml or lower		IL-6 above 10 pg/ml			IL-6 10 pg/ml or lower		IL-6 above 10 pg/ml		
IL-6 hours after admission [pg/ml]	3.42	1.33	3.82	1.40	0.34	3.45	1.06	3.73	0.79	0.38	3.06	0.64	3.09	0.30	0.87
IL-6 2nd day after admission [pg/ml]	3.41	1.33	3.76	1.39	0.21	3.44	1.06	3.64	1.04	0.37	3.07	0.65	3.00	0.29	0.62
IL-6 4 days after admission [pg/ml]	3.39	1.34	3.85	1.35	0.10	3.45	1.06	3.54	0.99	0.68	3.06	0.65	3.08	0.48	0.89
IL-6 1 week after admission [pg/ml]	3.42	1.38	3.78	1.25	0.21	3.44	1.09	3.52	0.98	0.73	3.07	0.67	3.07	0.47	0.95
IL-6 2 weeks after admission [pg/ml]	3.44	1.37	3.74	1.24	0.36	3.43	1.05	3.53	1.02	0.71	3.09	0.71	3.05	0.23	0.83

IL-6 – interleukin-6; pg/ml – picograms per milliliter; SD – standard deviation; p – significance level; x – mean

Data are presented as the mean and standard deviation. The effective sample size and subgroup denominators are reported for each biomarker time point. P values are descriptive because no formal correction for multiple comparisons was applied.
The IL-6 threshold above 10 pg/mL was used for exploratory threshold-based analyses and should not be interpreted as a standalone diagnosis of acute infection.

Table S10. Selected unadjusted associations between inflammatory markers and PICSQ severity at 3 months.

Marker	Endpoint	N	Mean ± SD in the reference group	N in the elevated marker group	Mean ± SD in the reference group	Difference of means	95% CI	p	Hedges g
CRP >5 mg/l after 1 week	PICSQ Psychological Domain (3 months)	216	3.06 ± 1.03	31	3.45 ± 0.93	0.40	0.03–0.76	0.039	0.39
PCT ≥2 ng/mL on day 4	PICSQ Total Domain (3 months)	245	11.44 ± 1.09	22	11.91 ± 0.61	0.46	0.16–0.76	0.015	0.44
PCT ≥2 ng/mL on day 4	PICSQ Psychological Domain (3 months)	245	3.05 ± 1.03	22	3.55 ± 0.91	0.50	0.07–0.92	0.027	0.49
IL-6 >10 pg/ml on day 2	PICSQ Psychological Domain (3 months)	241	3.04 ± 1.03	25	3.56 ± 0.82	0.52	0.16–0.88	0.013	0.51
IL-6 >10 pg/ml on day 4	PICSQ Psychological Domain (3 months)	239	3.04 ± 1.04	26	3.46 ± 0.86	0.42	0.05–0.79	0.041	0.41

CRP – C-reactive protein; PCT – procalcitonin; IL-6 – interleukin-6; PICS – Post-Intensive Care Syndrome; PICSQ – Post-Intensive Care Syndrome Questionnaire; SD – standard deviation; CI – confidence interval; Hedges g – standardized effect size corrected for small sample bias; N – number of patients; 3 months – assessment performed 3 months after ICU discharge

These are unadjusted exploratory comparisons. The adjusted analyses are presented in Supplementary Tables S11 and S12.

Table S11. Logistic regression models for higher psychological PICSQ domain severity at 3 months.

Marker	N	Percentage of patients with a score of ≥4 in the reference group	Percentage of patients with a score of ≥4 in the group with an elevated marker	OR	95% CI	p	aOR	95% CI	p
CRP >5 mg/l after 1 week	247	46.8%	67.7%	2.39	1.08-5.32	0.032	2.06	0.80-5.28	0.134
PCT ≥2 ng/mL on day 4	267	46.5%	72.7%	3.06	1.16-8.09	0.024	2.28	0.81-6.43	0.120
IL-6 >10 pg/ml on day 2	266	45.6%	76.0%	3.77	1.46-9.77	0.006	3.61	1.26-10.37	0.017
IL-6 >10 pg/ml on day 4	265	46.0%	69.2%	2.64	1.10-6.30	0.029	2.43	0.91-6.48	0.077

CRP – C-reactive protein; PCT – procalcitonin; IL-6 – interleukin-6; PICS – Post-Intensive Care Syndrome; PICSQ – Post-Intensive Care Syndrome Questionnaire; OR – odds ratio; aOR – adjusted odds ratio; CI – confidence interval; ICU – intensive care unit; N – number of patients; score ≥4 – clinically higher severity of psychological PICS domain at 3-month follow-up

Table S12 Multivariable linear regression models for selected PICSQ endpoints at 3 months.

Marker	Endpoint	N	β	95% CI	p	Adjusted R^2
CRP >5 mg/L after 1 week	PICSQ Psychological Domain (3 months)	247	0.30	- 0.15 to 0.74	0.196	0.011
PCT ≥2 ng/mL on day 4	PICSQ Total (3 months)	267	0.44	- 0.07 to 0.94	0.089	- 0.001
PCT ≥2 ng/mL on day 4	PICSQ Psychological Domain (3 months)	267	0.32	- 0.16 to 0.81	0.186	0.013
IL-6 >10 pg/ml on day 2	PICSQ Psychological Domain (3 months)	266	0.46	- 0.005 to 0.92	0.052	0.021
IL-6 >10 pg/ml on day 4	PICSQ Psychological Domain (3 months)	265	0.35	- 0.11 to 0.81	0.137	0.017

CRP – C-reactive protein; PCT – procalcitonin; IL-6 – interleukin-6; PICS – Post-Intensive Care Syndrome; PICSQ – Post-Intensive Care Syndrome Questionnaire; β – unstandardized regression coefficient; CI – confidence interval; adjusted R^2 – adjusted coefficient of determination; ICU – intensive care unit; N – number of patients; 3 months – assessment performed 3 months after ICU dischar

β represents the unstandardized regression coefficient. Adjusted models included age, sex, reason for ICU admission, and length of hospitalization. CRP, C-reactive protein; PCT, procalcitonin; IL-6, interleukin-6; PICSQ, Post Intensive Care Syndrome Questionnaire; CI, confidence interval; adjusted R^2, adjusted coefficient of determination; ICU, intensive care unit; N, number of patients; 3 months, assessment performed 3 months after ICU discharge.
